# Racial Differences in Ideal Cardiovascular Health Metrics Among Mississippi Adults, 2009 Mississippi Behavioral Risk Factor Surveillance System

**DOI:** 10.5888/pcd10.130201

**Published:** 2013-11-21

**Authors:** Vanessa L. Short, Abigail Gamble, Vincent Mendy

**Affiliations:** Author Affiliations: Abigail Gamble, University of Mississippi Medical Center, Jackson, Mississippi; Vincent Mendy, Mississippi State Department of Health, Jackson, Mississippi.

## Abstract

**Introduction:**

Cardiovascular disease is a leading cause of death and health disparities in Mississippi. Identifying populations with poor cardiovascular health may help direct interventions toward those populations disproportionately affected, which may ultimately increase cardiovascular health and decrease prominent disparities. Our objective was to assess racial differences in the prevalence of cardiovascular health metrics among Mississippi adults.

**Methods:**

We used data from the 2009 Mississippi Behavioral Risk Factor Surveillance System to determine age-standardized prevalence estimates and 95% confidence intervals of cardiovascular health metrics among 2,003 black and 5,125 white adults. Logistic regression models were used to evaluate the relationship between race and cardiovascular health metrics. The mean cardiovascular metrics score and percentage of the population with ideal and poor cardiovascular health were calculated by subgroup.

**Results:**

Approximately 1.3% of blacks and 2.6% of whites exhibited ideal levels of all 7 cardiovascular health metrics. The prevalence of 4 of the 7 cardiovascular health metrics was significantly lower among the total population of blacks than among whites, including a normal body mass index (20.8% vs 32.3%, *P* < .001), no history of diabetes (85.1% vs 91.3%, *P* < .001), no history of hypertension (53.9% vs 67.9%, *P* < .001), and physical activity (52.8% vs 62.2%, *P* < .001). The logistic regression models revealed significant race-by-sex interactions; differences between blacks and whites for normal body mass index, no history of diabetes mellitus, and no current smoking were found among women but not among men.

**Conclusion:**

Cardiovascular health is poor among Mississippi adults overall, and racial differences exist.

## Introduction

Cardiovascular disease (CVD), which includes heart disease and stroke, is a major public health issue. It is the leading cause of death in the United States (1) and is also the leading cause of health disparities in the nation, accounting for one-third of the excess mortality among non-Hispanic blacks compared with non-Hispanic whites (2,3). Healthy People 2020, a science-based, 10-year agenda for improving the health of all Americans, has an overarching goal to improve cardiovascular (CV) health and quality of life through prevention, detection, and treatment of risk factors for heart attack and stroke in the US population (4); the Division of Heart Disease and Stroke Prevention at the Centers for Disease Control and Prevention also works to improve CV health for all and reduce the burden associated with CVD (5). 

Recent recommendations from the American Heart Association (AHA) aim to improve CV health by encouraging the general population to meet 7 ideal CV health metrics (6). The 7 metrics include not smoking, being physically active, having normal blood pressure, having normal blood glucose levels, having normal total cholesterol levels, maintaining a healthy bodyweight, and having a healthful diet (6). Meeting a greater number of CV health metrics is associated with a lower risk of CVD and all-cause mortality (7).

Studies examining CV health metrics at the state level are limited; however, a recent analysis using Behavioral Risk Factor Surveillance System (BRFSS) data provided state-specific prevalence estimates of ideal (ideal metrics score of 7) and poor (ideal metrics score of 0–2) CV health and determined the mean CV health metrics score for all 50 US states and the District of Columbia (8). Overall, 3.3% of the adult U.S. population was in ideal CV health, and 9.9% was in poor CV health. Significant differences were observed by race: 9.2% of non‐Hispanic whites and 15.1% of non‐Hispanic blacks were in poor CV health (8). Compared to the other states, Mississippi derived the lowest mean CV score (score = 4.0; range across states, 4.0–4.7), the second lowest percentage of adults with all 7 ideal CV health metrics (1.5%; range across states, 1.2%– 6.9%), and the second highest percentage of adults with poor CV health (14.7%; range across states, 6.7%–16.2%) (8). These findings are not all that unexpected because CVD-related death rates in Mississippi are approximately 25% higher than national rates and are the highest in the United States for all races and both sexes (1). Moreover, marked racial health disparities exist; heart disease mortality rates among non-Hispanic blacks in Mississippi are approximately 20% higher than among non-Hispanic whites, and stroke mortality rates among non-Hispanic blacks are almost twice as high as non-Hispanic whites (9).

Theoretically, a difference in CVD health metrics may explain some of the observed disproportions. Nationally, disparities in CVD risk factors remain pervasive. For example, blacks are more likely than other racial groups to have adverse CVD risk factor profiles (10), to have higher prevalence and poorer control of hypertension (11-13), and to be obese (14). However, to our knowledge, no study has examined racial differences in CV health metrics among Mississippians. Identifying populations at high risk may help inform public health policy and direct intervention efforts toward those populations disproportionately affected, which may ultimately decrease CV-associated disease and death and decrease prominent health disparities. Therefore, we sought to assess racial differences in the prevalence of ideal CV health metrics among Mississippi adults.

## Methods

### Data source

Data from the 2009 Mississippi BRFSS were used for this analysis, because this was the most recent year with variables representing all 7 CV health metrics. The BRFSS is an ongoing, state-based, random digit-dialed telephone survey of noninstitutionalized adults aged 18 years or older. The survey collects self-reported information on health risk behaviors, preventive health practices, and health care coverage primarily related to chronic disease. The BRFSS is conducted in all 50 states, the District of Columbia, and 3 US territories (Puerto Rico, US Virgin Islands, and Guam). The BRFSS study has been approved by Human Research Review Boards from state departments of health. Further information on BRFSS is available at http://www.cdc.gov/brfss.

### Study population

Analyses were restricted to non-Hispanic blacks and non-Hispanic whites residing in Mississippi at the time of the survey; these racial/ethnic groups constitute more than 97% of the state’s population (15). We excluded participants who reported a history of coronary heart disease or stroke; those with missing information on sex, age, or race; and those without completed information on all 7 CV health metrics. Participants with a self-reported body mass index (BMI) under 18 kg/m^2^ were also excluded. Therefore, the final sample included 2,003 non-Hispanic black and 5,125 non-Hispanic white participants.

### Cardiovascular health metrics

Cardiovascular health metrics included self-reported measures of tobacco use, fruit and vegetable consumption, BMI, physical activity, diabetes, high blood pressure, and high blood cholesterol. Current smoking and history of diabetes, high blood pressure, and high blood cholesterol were coded as yes or no. BMI was coded normal (18.5–24.9 kg/m^2^) or above normal (≥25 kg/m^2^). Fruit and vegetable consumption was coded as 5 or more fruits and vegetables per day or fewer than 5 fruits or vegetables per day. Although the AHA’s metric for healthful diet is based on additional components, including whole grains, sodium, sugar-sweetened beverages, and fish, this score was not used in our analysis because these components are not assessed in BRFSS. Physical activity was coded as “meets the recommended level of physical activity per week” (participating in ≥30 min/d moderate physical activity for ≥5 d/wk, or ≥20 min/d vigorous physical activity on ≥3 d/wk) or does not meet the recommended level of physical activity per week (http://www.health.gov/paguidelines). Ideal levels of the CV health metrics were no current smoking, normal BMI, no history of diabetes, no history of hypertension, no history of high blood cholesterol, meets the recommended level of physical activity per week, and consumes 5 or more fruits and vegetables per day.

### Sociodemographic characteristics

Respondents were classified on the basis of self-reported information as non-Hispanic black or non-Hispanic white. Sociodemographic variables were assessed and considered as potential control variables in multivariate logistic models. Variables included self-reported age in years (18–34, 35–54, 55–64 and ≥65), education (less than high school, high school, more than high school), employment (employed, not employed), annual household income (categorized as a 4-level variable for annual household income, ranging from < $15,000 to ≥$50,000 or more), and having health care coverage (yes, no).

### Statistical analyses

Prevalence estimates and 95% confidence intervals (CIs) were calculated for sociodemographic characteristics and CV health metrics among racial groups. To allow for comparisons between groups, prevalence estimates of the CV health metrics were age-adjusted to the 2000 US census population (by using age distributions 18–24, 25–44, 45–64 and ≥65 y). The χ^2^ test was used to evaluate sociodemographic differences between blacks and whites.

Analyses were performed to compare blacks and whites in terms of CV health metrics. Logistic regression models were used to examine race differences as well as race-by-sex interactions on CV health metrics controlling for age, education, employment, and health care coverage. Income was not included because of the large percentage (12%) of respondents missing data on income. Results were expressed as odds ratios along with their corresponding 95% CIs and *P* values.

For each racial/ethnic group, we also calculated the percentage of the population with each ideal CV metrics score, defined as the number of ideal metrics (possible score of 0–7); the percentage of the population with ideal CV health (ie, ideal metrics score of 7); the percentage of the population with poor CV health (ie, ideal metrics score of 0, 1, or 2); and the mean ideal metrics score. Our definitions of ideal and poor CV health are consistent with AHA guidelines (6) and other published reports (8). The χ^2^ test and analysis of variance were used to evaluate differences between blacks and whites.

Data were analyzed using SAS version 9.3 (SAS Institute Inc, Cary, North Carolina) to adjust for the disproportionate stratified sampling design of BRFSS and were weighted by using post-stratification methods to allow for generalization of findings to the entire Mississippi adult population (16). *P* values of *P *< .05 were considered to be significant.

## Results

Compared with whites, blacks were generally younger and less educated, had higher rates of unemployment, had lower household incomes, and had lower rates of health care coverage (Table 1). Significant differences between blacks and whites were found in 5 of the 7 CV health metrics (Table 2). Compared with whites, blacks had a significantly lower prevalence of normal BMI (20.8% vs 32.3%, *P* < .001), no history of diabetes (85.1% vs 91.3%, *P* < .001), no history of hypertension (53.9% vs 67.9%, *P* < .001), and physical activity (52.8% vs 62.2%, *P* < .001). The prevalence of no history of high blood cholesterol was significantly higher among blacks than whites (66.3% vs 64.3%, *P* = .0003). Similar results were observed among women. Among men, the prevalence of physical activity (59.1% vs 69.6%, *P* = .003) and current smoking (72.1% vs 79.7%, *P* = .003) was significantly lower for blacks than for whites, whereas the prevalence of no history of blood cholesterol was significantly higher for blacks than for whites (66.5% vs 63.4%, *P* = .01).

**Table 1:** Sociodemographic Characteristics of Black and White Adults in Mississippi, Behavioral Risk Factor Surveillance System, 2009

Characteristic	Blacks^a^, %^b^ (95% CI)	Whites^a^, %^b^ (95% CI)	*P* Value^c^
**Sex**			
Male	45.9 (42.4–49.3)	47.5 (5.5–49.6)	.41
Female	54.1 (50.7–57.6)	52.5 (45.4–54.4)	
**Age, y**			
18–24	10.5 (7.3–13.6)	4.7 (3.2–6.1)	<.001
25–44	40.1 (36.7–43.6)	31.2 (29.1–33.3)	
45–64	35.0 (32.1–37.9)	43.3 (41.4–45.2)	
≥65	14.4 (12.8–15.9)	20.8 (19.6–22.0)	
**Education**			
<High school	19.6 (16.9–22.4)	8.5 (7.4–9.6)	<.001
High school	31.1 (28.0–34.1)	26.2 (24.5–27.8)	
>High school	49.3 (45.9–52.6)	65.3 (63.4–67.1)	
**Employment**			
Employed	49.1 (47.7–54.1)	54.2 (52.3–56.0)	.006
Not employed	50.9 (45.9–52.3)	45.8 (43.9–47.6)	
**Household income, $**			
<15,000	24.1 (21.4–26.8)	6.6 (5.7–7.4)	<.001
15,000–34,999	42.3 (38.9–45.8)	23.6 (21.8–35.4)	
35,000–49,999	13.0 (10.4–15.4)	15.8 (14.3–17.3)	
≥50,000	20.6 (17.7–23.4)	54.0 (51.9–56.1)	
**Health care coverage**			
Yes	76.4 (73.1–79.6)	90.2 (88.7–91.6)	<.001
No	23.6 (20.3–26.9)	9.8 (8.4–11.3)	

Abbreviations: CI, confidence interval.

a Sample sizes before weighting: blacks = 2,003; whites = 5,125.

b Weighted prevalence and 95% CIs.

c *P* values calculated by χ^2^ test.

**Table 2:** Age-Standardized Prevalence Estimates of Cardiovascular Health Metrics for Black and White Adults in Mississippi, Behavioral Risk Factor Surveillance System, 2009

Cardiovascular Health Metrics	Blacks^a^, % (95% CI)^b^	Whites^a^, % (95% CI)^b^	*P* Value^c^
**Normal BMI (18.5–24.9 kg/m^2^)**			
Overall	20.8 (17.8–23.8)	32.3 (29.6–35.0)	<.001
Male	24.2 (19.2–29.2)	22.0 (18.2–25.7)	.23
Female	17.9 (14.4–21.5)	42.2 (38.6–45.7)	<.001
**No history of diabetes**			
Overall	85.1 (83.5–86.8)	91.3 (90.4–92.2)	<.001
Male	87.4 (84.6–90.2)	90.7 (89.3–92.2)	.34
Female	83.7 (81.8–85.6)	91.8 (90.6–92.9)	<.001
**No history of hypertension**			
Overall	53.9 (50.9–56.8)	67.9 (65.7–70.2)	<.001
Male	57.3 (52.6–62.1)	66.0 (62.5–69.4)	.17
Female	51.3 (48.2–54.4)	70.0 (67.4–72.6)	<.001
**No history of high blood cholesterol**			
Overall	66.3 (63.7–68.9)	64.3 (62.2–66.3)	<.001
Male	66.5 (62.0–71.0)	63.4 (60.3–66.5)	.01
Female	66.0 (63.1–68.9)	65.2 (62.7–67.8)	.009
**No current smoking**			
Overall	79.6 (76.7–82.5)	80.2 (77.9–82.5)	.38
Male	72.1 (66.8–77.4)	79.7 (76.2–83.3)	.003
Female	85.8 (83.1–88.5)	80.8 (78.1–83.6)	.009
**Physically active^d^**			
Overall	52.8 (49.5–56.2)	62.2 (59.7–64.8)	<.001
Male	59.1 (53.5–64.7)	69.6 (65.8–73.4)	.003
Female	47.8 (43.9–51.6)	55.6 (52.6–58.6)	.031
**Consumes 5 or more fruits and vegetables/day**			
Overall	18.0 (15.2–20.8)	20.0 (17.7–22.3)	.38
Male	15.2 (10.7–19.7)	17.1 (13.6–20.5)	.74
Female	20.7 (16.9–24.4)	22.5 (19.6–25.4)	.30

Abbreviations: BMI, body mass index; CI, confidence interval.

a Sample sizes before weighting: blacks = 2,003; whites = 5,125.

b Weighted prevalence and 95% CIs.

c Calculated by χ^2^ test.

d Meets the 2008 recommended level of physical activity (http://www.health.gov/paguidelines).

The logistic regression models revealed significant race-by-sex interactions. The odds of normal BMI (*P* < .001), no history of diabetes (*P* = .005), no history of hypertension (*P* < .001), and no current smoking (*P* < .001) for blacks and whites differed by sex (Table 3). Significant differences between blacks and whites for normal BMI, no history of diabetes, and no current smoking were found among women but not among men. Compared with white women, black women were less likely to report a normal BMI (adjusted odds ratio [AOR] 0.29; 95% CI, 0.23–0.38) and no history of diabetes (AOR 0.44; 95% CI, 0.36–0.56). The odds of no current smoking were higher for black women than for white women (AOR 1.94; 95% CI, 1.48–2.53). Black women were about 63% less likely to report no history of hypertension than white women (AOR, 0.37; 95% CI, 0.30–0.45), whereas black men were about 32% less likely to report no history of hypertension than white men (AOR 0.68; 95% CI, 0.52–0.89).

**Table 3:** Comparison of Ideal Cardiovascular Health Metrics Among Mississippi Adults, Race Differences by Sex^a^, Behavioral Risk Factor Surveillance System, 2009

Cardiovascular Health Metrics	Unadjusted		Adjusted		*P* Value^c^, Race by Sex, Effect Modification,
	Males, Black vs White, OR (95% CI)	Females, Black vs White, OR (95% CI)	Males, Black vs White, AOR^b^ (95% CI)	Females, Black vs White, AOR^b^ (95% CI)	
Normal BMI (18.5–24.9 kg/m^2^)	1.22 (0.88–1.69)	0.30 (0.24–0.38)	1.22 (0.89–1.67)	0.29 (0.23–0.38)	<.001
No history of diabetes	0.85 (0.61–1.18)	0.55 (0.45–0.68	0.80 (0.56–1.14)	0.44 (0.36–0.56)	.005
No history of hypertension	0.83 (0.64–1.01)	0.55 (0.46–0.66)	0.68 (0.52–0.89)	0.37 (0.30–0.45)	<.001
No history of high blood cholesterol	1.40 (1.08–1.82)	1.26 (1.06–1.50)	1.18 (0.90–1.54)	1.03 (0.86–1.23)	.40
No current smoking	0.62 (0.45–0.85)	1.38 (1.09–1.76)	0.92 (0.65–1.28)	1.94 (1.48–2.53)	<.001
Physically active^d^	0.67 (0.51–0.87)	0.82 (0.68–0.98)	0.62 (0.47–0.81)	0.74 (0.61–0.88)	.30
Consumes 5 or more fruits and vegetables per day	0.93 (0.63–1.38)	0.88 (0.70–1.12)	1.03 (0.68–1.56)	0.91 (0.72–1.16)	.61

Abbreviations: AOR, adjusted odds ratio; CI, confidence interval; OR, odds ratio; BMI, body mass index.

a Sample sizes before weighting: black females, n = 1,413; black males, n = 590; white females, n = 3,297; white males, n = 1,828.

b Adjusted for age, education, employment, and health care coverage; whites as the referent.

c Calculated by χ^2^ test.

d Meets the 2008 recommended level of physical activity (http://www.health.gov/paguidelines).

We found no significant sex and race interaction effects for history of high blood cholesterol, physical activity, or fruit and vegetable consumption. After controlling for age, sex, education, employment, and health care coverage, blacks were less likely to be physically active than whites (AOR 0.68; 95% CI 0.58–0.80); we found no significant difference between blacks and whites for history of high blood cholesterol (AOR 1.09, 95% CI 0.93–1.28) or fruit and vegetable consumption (AOR 0.96; 95% CI 0.76–1.19).

Only 1.3% of blacks and 2.6% of whites had ideal levels of all 7 CV health metrics (Figure), whereas approximately 21% (95% CI, 18.7–23.1) of blacks and 14% (95% CI, 13.0–15.3) of whites had poor CV health. Compared with whites, blacks had a significantly lower mean CV health metrics score (3.73 vs 4.02, *P* < .001). The distribution of the number of ideal CV health components according to sex was fairly similar when examined across racial groups; white women had the highest mean ideal CV health metrics score (4.08), and black women had the lowest mean ideal CV metric score (3.66).

**Figure:**
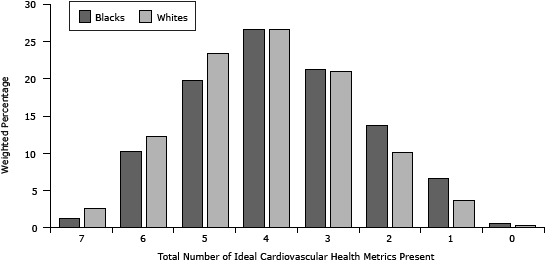
Distribution of number of ideal cardiovascular health metrics present by race, Mississippi Behavioral Risk Factor Surveillance System, 2009. Mean number of ideal cardiovascular health metrics present: blacks, 3.73 (95% CI, 3.63–3.83); whites, 4.02 (95% CI, 3.97–4.07); analysis of variance *P* value < .0001. Sample sizes before weighting: blacks = 2,003; whites = 5,125. Percentages refer to weighted prevalence. **Table:** 

Total Number of Ideal Cardiovascular Health Metrics Present	Blacks, %	Whites, %
7	1.3	2.6
6	10.2	12.3
5	19.7	23.4
4	26.5	26.6
3	21.2	20.9
2	13.7	10.1
1	6.6	3.7
0	0.62	0.37

## Discussion

In Mississippi, the conditions in which people live and the systems in place to manage disease and illness likely contribute to the state’s poor CV health and the racial disparity gaps in ideal CV health. An annual medical visit to a primary care physician offers an opportunity to address health risk factors and behaviors. Physicians and other health care professionals have an important opportunity to communicate to their patients the serious health consequences associated with excess body weight, smoking, physical inactivity and poor diet. However, much of Mississippi is considered medically underserved (17) with limited availability of health care professionals, including primary care physicians. This presents 2 important challenges. First, many Mississippians may only interact with the health care system once they experience more serious and symptomatic conditions requiring secondary or tertiary treatment provided by a specialist or an emergency department. Hence, in many of these instances, poor CV health and CVD is likely present. Second, those Mississippians who lack access to preventive care because of health care provider shortages or lack of health care coverage are less likely to receive important health messages related to chronic disease prevention. This lack of access to primary health care may contribute to the low prevalence of ideal health behaviors, such as not smoking, physical activity, proper diet, and weight management in the study population as a whole.

An overarching goal of *Healthy People 2020* is to achieve health equity, eliminate disparities, and improve the health of all groups (4). Health disparities are defined as differences in health outcomes between groups that reflect social inequalities (18). The risk for unhealthy behaviors, reduced access to health care, poor quality of care, illness, and death increases with decreasing socioeconomic status (19,20). Education and income are 2 indicators commonly used to measure the influence of socioeconomic conditions on health (21); education is a strong predictor of employment and income, and income directly measures material resources, which influence health through a direct effect on living conditions (22) and subsequent health behaviors. In comparison to white adults in our study population, more black adults were unemployed, were less educated, were less likely to have health care coverage, and had lower household incomes. Hence, social determinants of health may explain a portion of the differences observed between black and white adults and may provide insight into health care access and use.

Environmental access to and availability of healthy food outlets and physical activity–friendly resources are demonstrable social determinants of health. Residential segregation according to income has increased in recent decades (23), and as a result, minorities may be more likely than whites to live in distressed and disadvantaged neighborhoods. In our study population, black adults may have been more likely to reside in communities that offered less support for healthy lifestyle behaviors compared with their white counterparts. Unhealthy and inequitable living environments combined with less education and lower income have the potential to create ideal circumstances for increased exposure to CVD risk factors and, conceivably, to foster a culture characterized by unhealthy living practices.

Cultural values, beliefs, and attitudes about what is considered healthy may also contribute to some of the differences found between racial groups. For example, studies have documented that a larger body size may be viewed more positively by black women (24,25) and that among overweight women, blacks tend to report less body dissatisfaction than whites. It could be that the disparities we found in our analysis were mainly driven by differences between black women and white women because fewer differences were found between black men and white men. Some variations in race-by-sex interaction terms were found to be significant. These findings could have implications for interventions and public health messaging that targets specific populations. Future studies should consider examining these race–sex differences further.

Our study has several limitations. First, although surveys such as BRFSS provide key state data on prevalence of risk factors, such surveys focus on a broad spectrum of diseases and do not describe all CVD risk factors. Nor does BRFSS capture environmental perceptions or existing policies and environmental features that support or deter healthful choices. Second, BRFSS data are self-reported, and the survey does not allow for direct measures. Consequently, risk factors may be over- or underreported because of social desirability bias (eg, smoking) or recall bias (eg, health care provider, diagnosed medical condition). Third, there may have been sampling bias resulting from random-digit dialing, and thus, an underrepresentation of subpopulations that are not likely to have a home telephone. In the second half of 2009, 35.1% of Mississippians lived in households with only cellular telephone service (26), and in general, US adults living in cellular telephone–only households tend to be younger, have lower incomes, and be members of minority populations (27). Finally, Mississippi is among the poorest states in the country, and thus, poor economic conditions likely influence health care coverage and use of preventive health services and contribute to the burden of poor health status among residents. Although we did not assess differences by health care coverage or household income, future studies should consider examining differences by these socioeconomic indicators.

Notwithstanding these limitations, our results provide information on state-specific needs and could be used to determine priorities for public health messages and to inform evidence-based, culturally-appropriate intervention strategies aimed at reducing and ultimately eliminating CVD-related health disparities in Mississippi. However, before considering individual-level interventions, practitioners should consider environmental and policy approaches. A substantial body of evidence shows that environmental and policy interventions are effective in preventing chronic disease risk factors (28). Environmental and policy approaches that aim to reduce exposure to the complex interaction of risk factors linked to the development and onset of chronic disease are designed to provide opportunities, support, and cues to help people develop and maintain healthful lifestyle behaviors (28). A first step in these efforts is to document the extent and nature of health disparities (29) and to conduct an assessment of the local context to understand how intervention strategies apply within populations with large health disparities (28). With this knowledge base, public health practitioners and researchers will be prepared to engage communities in a collaborative and community-driven approach to create healthy change.

To our knowledge, this is the first study to assess racial differences in the prevalence of ideal CV health metrics among Mississippi adults using a population-based, representative sample. The prevalence of ideal levels of CV health characteristics is low among Mississippi adults, and racial differences exist. Strategies to improve CV health should recognize these disparities to improve their effectiveness.
